# Technology-Driven Dyadic Interaction Support for Community-Dwelling People With Dementia and Family Caregivers

**DOI:** 10.1093/geroni/igab046.2248

**Published:** 2021-12-17

**Authors:** Karin Wolf-Ostermann, Lars Steinert, Tanja Shultz, Viktoria Hoel

**Affiliations:** 1 University of Bremen, University of Bremen, Bremen, Germany; 2 University of Bremen, Bremen, Bremen, Germany

## Abstract

People with dementia and their family caregivers struggling with the impacts of the condition on cognitive abilities, experience deterred social interactions and strained relationships. Technology can potentially sustain the relationship by engaging dyads in joint activities and supporting their interaction. This study aimed to evaluate the impact of a tablet-based activation system, I-CARE, specifically designed to engage people with dementia in meaningful activities. In this intervention, community-dwelling people with dementia and their family caregiver engaged in joint activities supported by the I-CARE system. Quantitative measures on quality of life, relationship quality and caregiver burden are collected, while semi-structured interviews explore the impact of Covid-19, as well as what motivates the participants to invite technology into their dyadic interactions. Our findings provide important insight in how technology can support social health and relationship sustenance of dyads living with dementia, and what implications Covid-19 has for their social participation in society.

